# Low mucosal-associated invariant T-cell number in peripheral blood of patients with immune thrombocytopenia and their response to prednisolone

**DOI:** 10.1371/journal.pone.0207149

**Published:** 2018-11-08

**Authors:** Takaaki Maekawa, Yukiko Osawa, Yosuke Okada, Noriaki Tachi, Masahiro Teramoto, Toshikuni Kawamura, Toshikatsu Horiuchi, Shoichiro Kato, Ayako Kobayashi, Shinichi Kobayashi, Ken Sato, Fumihiko Kimura

**Affiliations:** Division of Hematology, Department of Internal Medicine, National Defense Medical College, Tokorozawa, Saitama, Japan; Karolinska Institutet Department of Medicine Solna, SWEDEN

## Abstract

Mucosal-associated invariant T (MAIT) cells help protect against certain infections and are related to some autoimmune diseases. Immune thrombocytopenia (ITP) is a relatively rare hematological autoimmune disease associated with low platelet count. We designed a cross-sectional study wherein we examined peripheral blood samples of patients with ITP for the number of MAIT cells (CD3^+^TCR-Vα7.2^+^CD161^+^IL-18Rα^+^ lymphocytes) and their CD4/8 subsets (by flow cytometry) and levels of cytokines (by multiplex assays). The study cohort included 18 patients with ITP and 20 healthy controls (HCs). We first compared the number of MAIT cells between HCs and patients with ITP and then performed subgroup analysis in patients with ITP. The number of total MAIT cells in patients with ITP was significantly lower than that in HCs (*p* < 0.0001), and the CD4^−^CD8^+^ subset of MAIT cells showed the same trend. Moreover, patients with ITP refractory to prednisolone exhibited a significantly lower number of total MAIT and CD4^−^CD8^+^ MAIT cells than patients sensitive to prednisolone. The number of total MAIT and CD4^−^CD8^+^ MAIT cells was not correlated with the response to thrombopoietin receptor agonist treatment or with *Helicobacter pylori* infection. We found no relation between cytokine levels and response to prednisolone treatment, although the levels of IP-10 and RANTES showed a correlation with the number of total MAIT and CD4^−^CD8^+^ MAIT cells. In conclusion, total MAIT and CD4^−^CD8^+^ MAIT cells in peripheral blood were decreased in patients with ITP, correlating with their response to prednisolone.

## Introduction

Mucosal-associated invariant T (MAIT) cells produce various cytokines and control various types of immunoreactions. They are innate T lymphocytes that connect innate and acquired immunities. MAIT cells are abundantly present in humans; they comprise 20%–50% of the T cells in the liver and 1%–10% of the intestinal mucosa lamina propria lymphocytes and the peripheral blood mononuclear cells (PBMCs) [1–2]. The ability of MAIT cells to recognize microbial derived vitamin B metabolites presented on the major histocompatibility complex class I-related molecule (MR1) enables them to detect various species of bacteria and yeast in vitro and in vivo [3–4]. In addition, MAIT cells are also activated by pro-inflammatory cytokines, such as IL-12 and IL-18, in a T-cell receptor (TCR)-independent manner [5]. Once activated, MAIT cells secrete pro-inflammatory cytokines, such as IL-17 and IFNγ, and exhibit cytotoxic effects [1, 6]. MAIT cells express the specific invariant T-cell receptor alpha chain (TCR-Vα7.2), CD161 (one of the C-type lectins), and interleukin-18-receptor alpha chain (IL-18Rα), which are used as specific surface markers [4, 7]. The majority of MAIT cells are CD4^−^CD8^+^, followed by CD4^−^CD8^−^ [4].

MAIT cells may play a role in the onset and progress of some inflammatory and autoimmune diseases. Ninety percent of the CD8^+^CD161^high^ T cells consist of MAIT-specific TCR-Vα7.2^+^ cells in human PBMCs [8]; they accumulate in inflammatory regions, such as the liver or joints, and are regarded as an onset factor for multiple sclerosis, in which case they can be found in active lesion sites [9–10]. MAIT cells are also found in lesion sites of patients with chronic inflammatory demyelinating polyneuritis [9]. Moreover, MAIT cells protect against drug-induced inflammatory tissue injuries, and this has led to the suggestion that MAIT cells play a role in some inflammatory bowel diseases represented by ulcerative colitis and Crohn’s disease [11–13]. Circulating MAIT cell levels are low in patients with systemic lupus erythematosus and rheumatoid arthritis [14] and also in patients with autoimmune liver diseases, where MAIT cell frequency decreases with fibrosis severity [15]. MAIT cells also help in regulating the immune response in allogeneic transplantations [16].

Autoimmunity mechanisms are part of the pathogenesis of several blood disorders, including autoimmune hemolytic anemia, aplastic anemia, and immune thrombocytopenia (ITP). ITP is an acquired immune disease characterized by isolated thrombocytopenia, identified by a peripheral blood (PB) platelet count of <100 × 10^9^ cells/L and the lack of an obvious cause for the thrombocytopenia [17]. The classical concept of ITP being caused by accelerated platelet destruction mediated by autoantibodies has shifted to a more complicated mechanism in which both reduced platelet production and T-cell-mediated effects play a role [17]. In addition, the relationship between *Helicobacter pylori* infection and ITP was initially described in 1998, and Stasi et al. demonstrated that the overall response rate of *H*. *pylori* eradication in ITP patients was 50.3% [18]. In contrast, corticosteroids have long been used as a first-line therapy for patients with ITP and are thought to work by globally influencing the immune system to functionally suppress both T and B cell reactivities [19]. However, some patients with ITP are refractory to corticosteroid treatment, the cause of which remains unclear. Incidentally, circulating Th17 cell levels have been associated with sensitivity to corticosteroids in patients with ITP [20], and this suggests that T-cell abnormalities impact corticosteroid reactivity.

The analysis of MAIT cells may elucidate the pathogenesis of ITP, and MAIT cells could become an immunotherapy target; however, no study has verified the relationship between ITP and MAIT cells. Thus, the purpose of our study was to evaluate, for the first time, the quantitative changes of MAIT cells in patients with ITP, and we plan to will develop a new diagnostic method in the future.

## Materials and methods

### Human subjects

This study was performed in accordance with the guidelines of the National Defense Medical College ethics committee (Receipt number: 2341). Eighteen patients with ITP (14 women and 4 men; mean age, 68.1 years) seen during the observation period of 2015–2017 were enrolled after they provided written informed consent. All patients were treated with prednisolone or a thrombopoietin receptor (TPO-R) agonist or with both, and we tested them for *H*. *pylori* infection. We defined the treatment response according to the report from an international working group [17]. “Complete response” (CR) was defined as any platelet count of at least 100 × 10^9^ cells/L. “Response” (R) was defined as any platelet count between 30 and 100 × 10^9^ cells/L and at least doubling of the baseline count. “No response” (NR) was defined as any platelet count <30 × 10^9^ cells/L or less than doubling of the baseline count. “Loss of R” was defined as any platelet count <30 × 10^9^ cells/L or less than a two-fold increase of the baseline platelet count or bleeding after having had a response (R). We assessed the response to prednisolone throughout the observation period and defined the response to TPO-R agonists based on the maximum response. For hematological evaluations, PB samples were collected from all patients and from 20 healthy controls (HCs) (16 women and 4 men; mean age, 43.1 years) after they provided written informed consent. The individual characteristics of HCs and patients with ITP are given in Table 1, and the course of treatment in individual ITP patients is given in S1 Fig. In addition, to assess whether prednisolone affected the number of MAIT cells in the PB, we conducted a follow-up survey on two prednisolone-treated patients with ITP after obtaining written informed consent. In these patients, the first PB samples were used in the analyses.

**Table 1:** Characteristics of healthy controls and patients with immune thrombocytopenia.

	Healthy controls	Patients with ITP
Number	20	18
Men/Women	4/16	4/14
Age (years), mean ± SD	43.1 ± 9.0	68.1 ± 13.8
Response to prednisolone treatment		
CR		2
R		5
NR		2
Loss of R		6
No prednisolone treatment		3
Response to TPO agonist treatment		
CR		11
R		5
No TPO agonist treatment		2
*Helicobacter pylori* infection		
No		9
Yes		9
Splenectomy		
No		16
Yes		2

Abbreviations: SD, standard deviation; CR, complete response; ITP, Immune thrombocytopenia; R, response; NR, no response; TPO, thrombopoietin.

### Flow cytometric analysis

Human PBMCs were collected from whole blood samples by the specific gravity centrifugal method using Lymphoprep’s protocol (1114547; Alere Technologies AS, Oslo, Norway). PBMCs were resuspended in 10 μL of FACS buffer (PBS with 2% FBS and 0.05% NaN_3_) containing FITC-conjugated anti-CD3 antibody (A07746; Beckman Coulter, Tokyo, Japan) or FITC-conjugated anti-CD8 antibody (130-080-601; Milteny Biotec, Bergish Gladbach, Germany), PE-conjugated anti-CD218a (IL-18Ra) antibody (130-101-657; Milteny Biotec) or PE-conjugated anti-CD4 antibody (R0805; Dako, Tokyo, Japan), APC-conjugated anti-CD161 antibody (130-098-908; Milteny Biotec), and VioBlue-conjugated anti-TCR-Vα7.2 antibody (130-100-211; Milteny Biotec). After incubating for 30 min on ice, the cells were washed twice and analyzed using BD FACS Aria III. We began by gating CD3^+^TCR-Vα7.2^+^ lymphocytes and then the CD161^+^IL-18Rα^+^ subset to define MAIT cells. To distinguish the CD4 CD8 subset of MAIT cells, we gated CD161^+^TCR-Vα7.2^+^ lymphocytes and then divided them into CD4^+^CD8^+^, CD4^+^CD8^−^, CD4^−^CD8^−^, and CD4^−^CD8^+^ subsets (S2 Fig). This gating strategy can accurately identify CD4^−^CD8^−^ and CD4^−^CD8^+^ subsets, but is not enough for CD4^+^CD8^+^ and CD4^+^CD8^−^ subsets because we did not use the MR1 tetramer—this is a limitation of this study. The absolute counts of MAIT cells in 1 ml of human PB were calculated using CountBright absolute counting beads (C36950; Thermo Fisher Scientific, Waltham, MA, USA).

### Immunoassays

Plasma samples of HCs (*n* = 3) and patients with ITP (*n* = 15) were stored at −20°C until analysis. The plasma levels of the following cytokines, chemokines, and growth factors were determined using Bio-Plex Pro Human Cytokine Assays (Bio-Rad Laboratories, Hercules, CA, USA): IL-1ß, IL-1Ra, IL-2, IL-4, IL-5, IL-6, IL-7, IL-8, IL-9, IL-10, IL-12, IL-13, IL-15, IL-17, Eotaxin, FGF, IFN-γ, Interferon-inducible protein 10 (IP-10), G-CSF, GM-CSF, MIP-1α, MCP-1, MIP-1ß, PDGF-BB, Regulated-upon-activation normal T cells expressed and secreted (RANTES), TNF-α, and VEGF. Bio-Plex Pro Assays are immunoassays formatted on magnetic beads that utilize principles similar to those of a sandwich ELISA. Briefly, capture antibodies against the biomarker of interest were covalently coupled to the beads. Biotinylated detection antibodies created the sandwich complexes, and the final detection complex was formed by the addition of a streptavidin–phycoerythrin conjugate, where phycoerythrin served as the fluorescent reporter. Reactions were read using a Luminex-based reader [21].

### Statistical analysis

Descriptive statistic results are reported as the mean ± standard deviation of data obtained from three individual experiments, unless specified otherwise. Statistical significance was determined using the Mann–Whitney U test or Steel–Dwass test. All p-values were two-sided, and values <0.05 were considered statistically significant. Data were plotted, and statistical analyses were performed using the GraphPad Prism 7.0 software (GraphPad Software, La Jolla, CA, USA).

## Results

### Circulating MAIT cell number and frequency within blood CD3^+^ T cells were significantly decreased in patients with ITP, and the CD4^−^CD8^+^subset was the most affected

**Fig 1:**
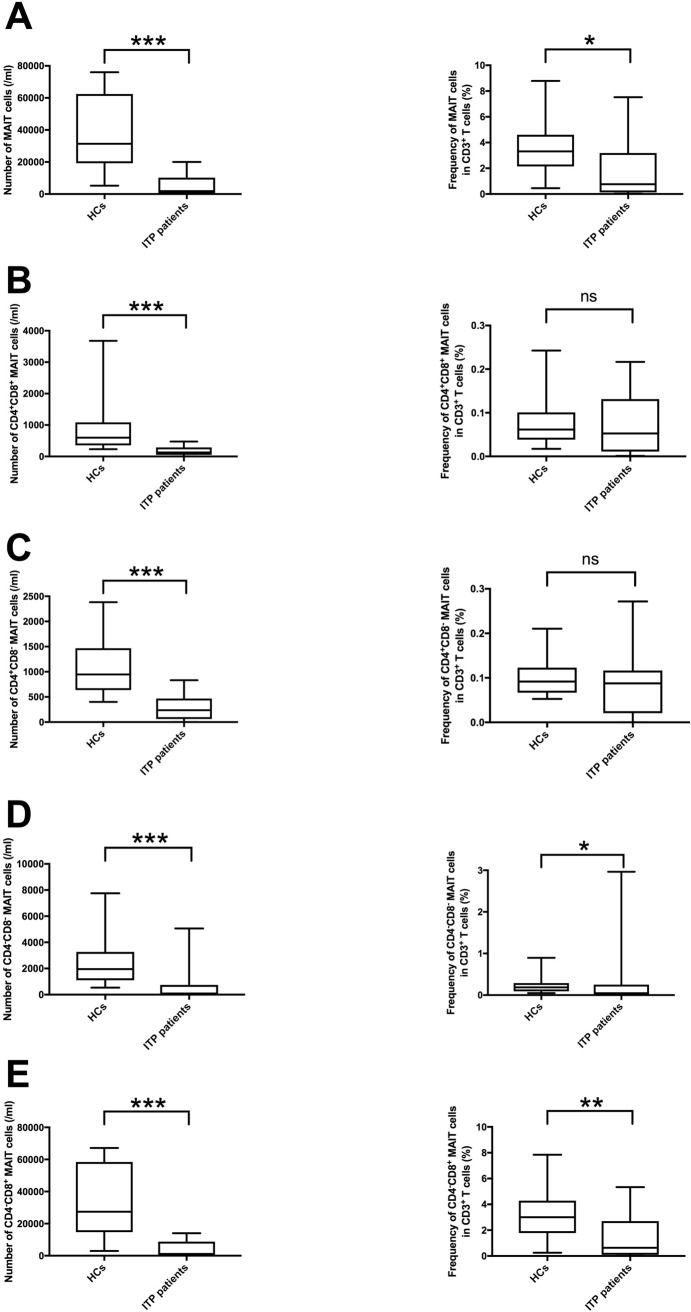
Number and rate in the blood CD3^+^ T cells of mucosal-associated invariant T (MAIT) cells in the peripheral blood of healthy controls (HCs) (*n* = 20) and patients with immune thrombocytopenia (ITP) (*n* = 18). **(A)** Number and frequency in the blood CD3^+^ T cells of total MAIT cells in the peripheral blood of HCs and patients with ITP. We defined the total MAIT cells by gating CD3^+^TCR-Vα7.2^+^ lymphocytes and then gating the CD161^+^IL-18Rα^+^ subset. The number of MAIT cells of patients was significantly lower than that of HCs. **(B)** Number and frequency in the blood CD3^+^ T cells of CD4^+^CD8^+^ MAIT cells subset in the peripheral blood of HCs and patients with ITP. To distinguish the CD4 CD8 subset of MAIT cells, we gated CD161^+^TCR-Vα7.2^+^ lymphocytes and after that divided them into CD4^+^CD8^+^, CD4^+^CD8^−^, CD4^−^CD8^−^, and CD4^−^CD8^+^ subsets. **(C)** Number and frequency in the blood CD3^+^ T cells of CD4^+^CD8^−^ MAIT cells subset in the peripheral blood of HCs and patients with ITP. **(D)** Number and frequency in blood CD3^+^ T cells of CD4^−^CD8^−^ MAIT cells subset in the peripheral blood of HCs and patients with ITP. **(E)** Number and frequency in the blood CD3^+^ T cells of CD4^−^CD8^+^ MAIT cells subset in the peripheral blood of HCs and patients with ITP. This subset showed the same trend as total MAIT cells. Statistical significance is calculated by the Mann–Whitney U test, *p < 0.05, **p < 0.005, ***p < 0.0001.

We measured the number of MAIT cells (CD3^+^TCR-Vα7.2^+^CD161^+^IL-18Rα^+^ lymphocytes) in the PB of patients with ITP and HCs by flow cytometry. We found that the number and frequency of total MAIT cells within blood CD3^+^ T cells in ITP patients were significantly lower than those in HCs (*p* < 0.0001 and *p* = 0.0123, respectively) (Fig 1A). In addition, the number of CD4^+^CD8^+^, CD4^+^CD8^−^, CD4^−^CD8^−^, and CD4^−^CD8^+^ subsets of MAIT cells in patients with ITP were significantly lower than those in HCs (*p* < 0.0001) (Fig 1B, 1C, 1D and 1E), and the frequency of CD4^−^CD8^−^ and CD4^−^CD8^+^ subsets of MAIT cells within blood CD3^+^ T cells were significantly lower than those in HCs (*p* = 0.0377 and *p* = 0.0048, respectively) (Fig 1D and 1E). Although both the number and frequency of total MAIT cells and CD4^−^CD8^+^ subsets of MAIT cells represented significant differences between HCs and ITP patients, the contrast was clearer in terms of number than of frequency. Due to these findings and the limitations of the flow cytometry gating strategy (the CD4^+^ subsets were not perfectly identified), we decided to perform the subsequent subgroup analyses using the number of total MAIT cells, including the CD4^−^CD8^+^ and CD4^−^CD8^−^ subsets of MAIT cells.

### Patients with ITP refractory to prednisolone treatment exhibited a significantly smaller number of total MAIT and CD4^−^CD8^+^ MAIT cells than those who responded to the treatment

**Fig 2:**
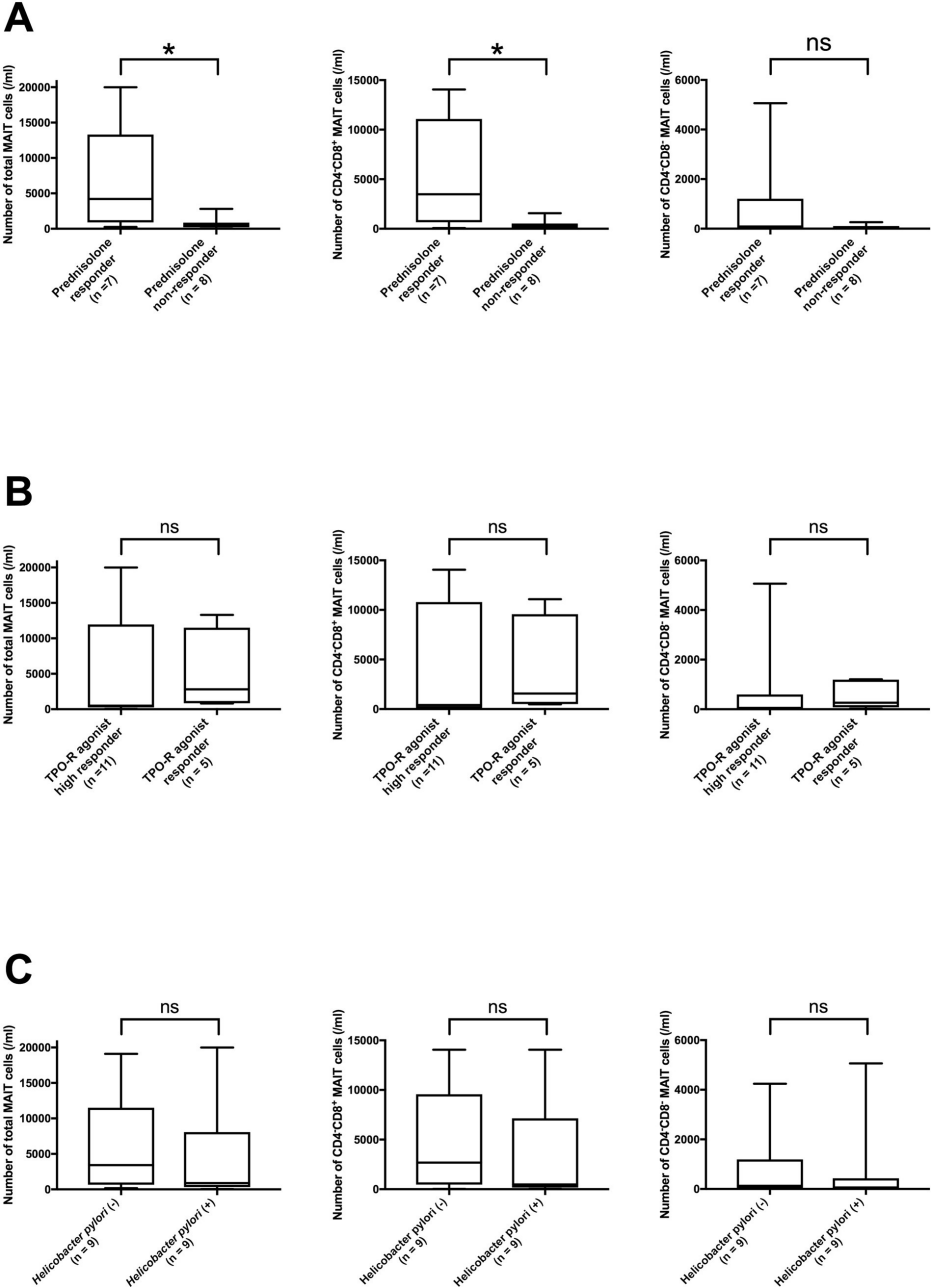
Comparison of the number of CD4^−^CD8^+^ MAIT cells between the subgroups of patients with ITP based on the response to treatment or the presence of *Helicobacter pylori* infection. **(A)** The number of total MAIT, CD4^−^CD8^+^ MAIT, and CD4^−^CD8^−^ MAIT cells in responders to prednisolone treatment [ITP patients achieving complete response (CR) and response (R)] (*n* = 7) and non-responders to prednisolone treatment [ITP patients exhibiting no response (NR) and loss of R] (*n* = 8). The non-responders exhibited a significantly lower number of total MAIT and CD4^−^CD8^+^ MAIT cells than the responders. **(B)** The number of total MAIT, CD4^−^CD8^+^ MAIT, and CD4^−^CD8^−^ MAIT cells in high responders to thrombopoietin receptor (TPO-R) agonist treatment [ITP patients achieving CR] (*n* = 11), and responders to TPO-R agonist treatment [ITP patients going no further than R] (*n* = 5). We found no significant changes in the number of total MAIT, CD4^−^CD8^+^ MAIT, or CD4^−^CD8^−^ MAIT cells between the subgroups. **(C)** The number of total MAIT, CD4^−^CD8^+^ MAIT, and CD4^−^CD8^−^ MAIT cells in patients with ITP with (*n* = 9, 8 of them were treated with prednisolone) or without (*n* = 9, seven of them were treated with prednisolone) *H*. *pylori* infection. We found no significant changes in the number of total MAIT, CD4^−^CD8^+^ MAIT, or CD4^−^CD8^−^ MAIT cells between the two subgroups. Statistical significance was calculated by the Mann–Whitney U test, *p < 0.05.

We compared the number of total MAIT, CD4^−^CD8^+^ MAIT, and CD4^−^CD8^−^ MAIT cells among patients with ITP on the basis of their response to treatment or the presence of *H*. *pylori* infection. We defined patients with sustained CR or R as responders to prednisolone treatment (*n* = 7) and those with NR or loss of R as non-responders to prednisolone treatment (*n* = 8). Our results showed that non-responders to prednisolone treatment exhibit a significantly lower number of total MAIT and CD4^−^CD8^+^ MAIT cells than responders to prednisolone treatment (*p* = 0.014 and *p* = 0.014, respectively) (Fig 2A).

In contrast, all patients treated with TPO-R agonist achieved CR or R, so we assessed their response based on the maximum response; 11 patients achieved CR (high responders to the TPO-R agonist treatment), and 5 patients went no further than R (responders to the TPO-R agonist treatment). We found no significant difference in the number of total MAIT, CD4^−^CD8^+^ MAIT, and CD4^−^CD8^−^ MAIT cells between the two subgroups (Fig 2B). The results of the comparison of the number of total MAIT, CD4^−^CD8^+^ MAIT, and CD4^−^CD8^−^ MAIT cells between patients with (*n* = 9, eight of them were treated with prednisolone) and without (*n* = 9, seven of them were treated with prednisolone) *H*. *pylori* infection showed no significant difference between the two subgroups (Fig 2C).

**Fig 3:**
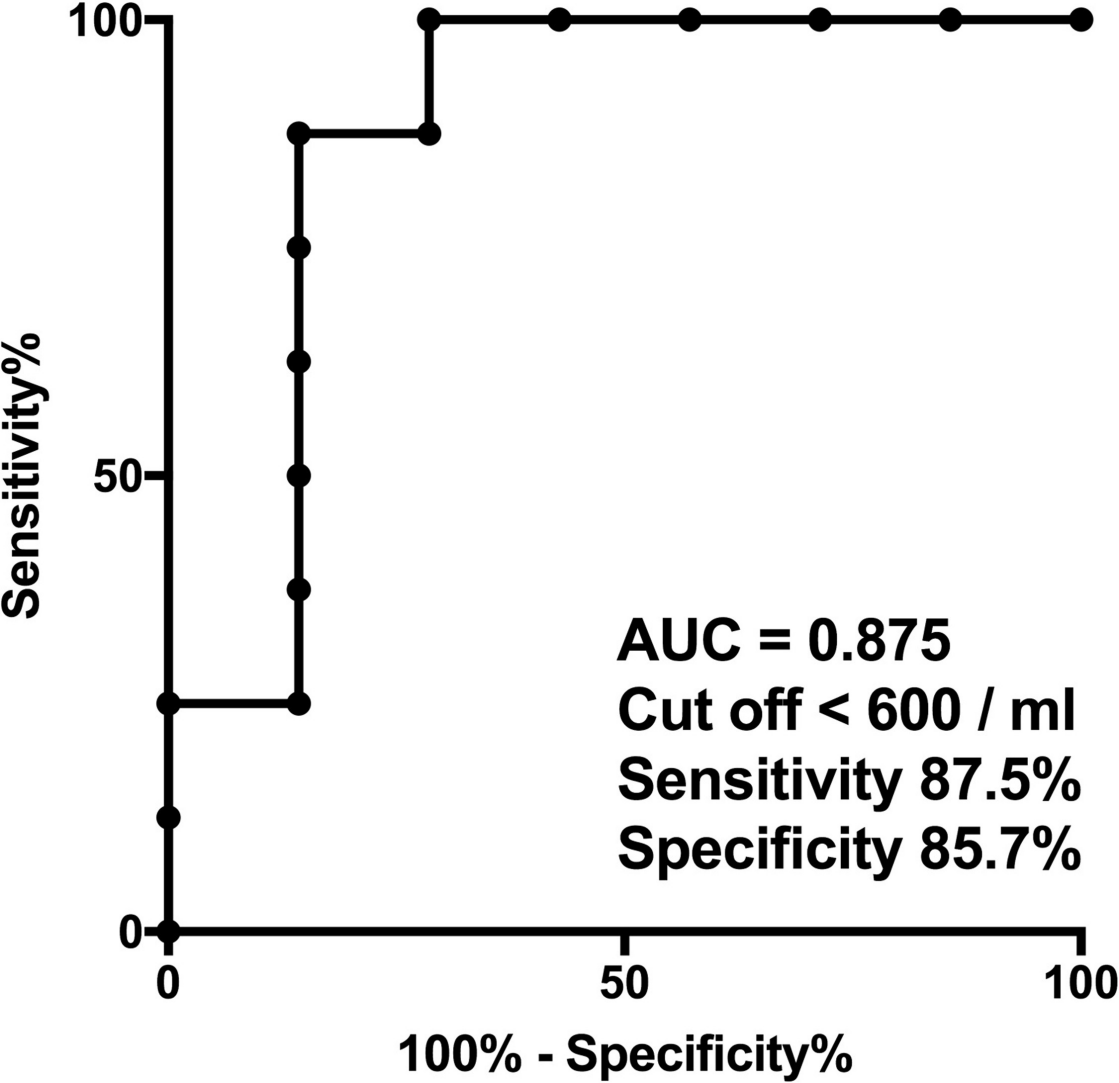
Receiver operating characteristic curve showing the cut-off value of the number of CD4^−^CD8^+^ MAIT cells in the peripheral blood for the corticosteroid-sensitive subgroup (CR and R) versus the corticosteroid-refractory subgroup (NR and loss of R) in patients with ITP. The area under the curve was 0.875, and a cut-off value <600 cells/ml signaled a sensitivity and specificity of refractoriness to corticosteroid treatment of 87.5% and 85.7%, respectively.

We drew receiver operating characteristic curves to obtain a cut-off of the number of CD4^−^CD8^+^ MAIT cells in PB for separating the responders to prednisolone treatment (CR and R) from the non-responders to prednisolone treatment (NR and loss of R). The area under the curve was 0.875, and by setting the cut-off of the number of CD4^−^CD8^+^ MAIT cells to a value <600 cells/ml, the sensitivity and specificity in classifying refractoriness to prednisolone treatment were 87.5% and 85.7%, respectively (Fig 3).

The effect of corticosteroids on MAIT cells is controversial. The number of MAIT cells in patients with systemic lupus erythematosus is not correlated with the use of corticosteroids, immunosuppressive agents, or disease-modifying antirheumatic drugs [14]. In addition, the frequency of MAIT cells in patients with multiple sclerosis significantly increased 2–3 months after steroid therapy [10]. In contrast, Hinks et al. demonstrated that the frequency of MAIT cells was suppressed after 7 days of treatment with 20 mg oral prednisolone in patients with asthma [22–24]. To examine the effect of prednisolone exposure in our study, we evaluated the correlations between MAIT cells and the duration of prednisolone treatment at the time of blood sample collection in patients with ITP. We excluded four patients whose prednisolone treatment was discontinued for more than 1 month before blood collection in this analysis. Results showed no correlation between the duration of prednisolone treatment and the number of MAIT cells as well as the frequency of MAIT cells in the CD3^+^ T cells (S3 Fig).

Furthermore, to assess whether prednisolone affected the number of MAIT cells in PB of ITP patients, we conducted a follow-up survey in two prednisolone-treated patients with ITP. One patient (responder to prednisolone treatment) was newly diagnosed during the observation period of our study, and the first PB sample of this patient was collected before corticosteroid initiation. The second and third PB samples were obtained 3 and 6 months after the corticosteroid treatment initiation. Our results indicate that the number of CD4^−^CD8^+^ MAIT cells after 3 and 6 months did not decrease compared with that in the first sample (S4 Fig). In contrast, the corticosteroid treatment of the other patient tested (non-responder to prednisolone treatment) was discontinued during the observation period. The first and second samples were collected 6 and 24 months after corticosteroid discontinuation, respectively. We observed that the number of CD4^−^CD8^+^ MAIT cells remained extremely low (considering that the range of the number of CD4^−^CD8^+^ MAIT cells in the PB of HCs is 20000–80000 cells/ml) even after 24 months post corticosteroid discontinuation (S4 Fig). Although our examinations included very few patients, our observations were in agreement with a report on the lack of correlation between the number of MAIT cells and the administration of steroids [14].

### Cytokine levels did not correlate with the response to corticosteroids, although IP-10 and RANTES showed a correlation with the number of CD4^−^CD8^+^ MAIT cells

**Fig 4:**
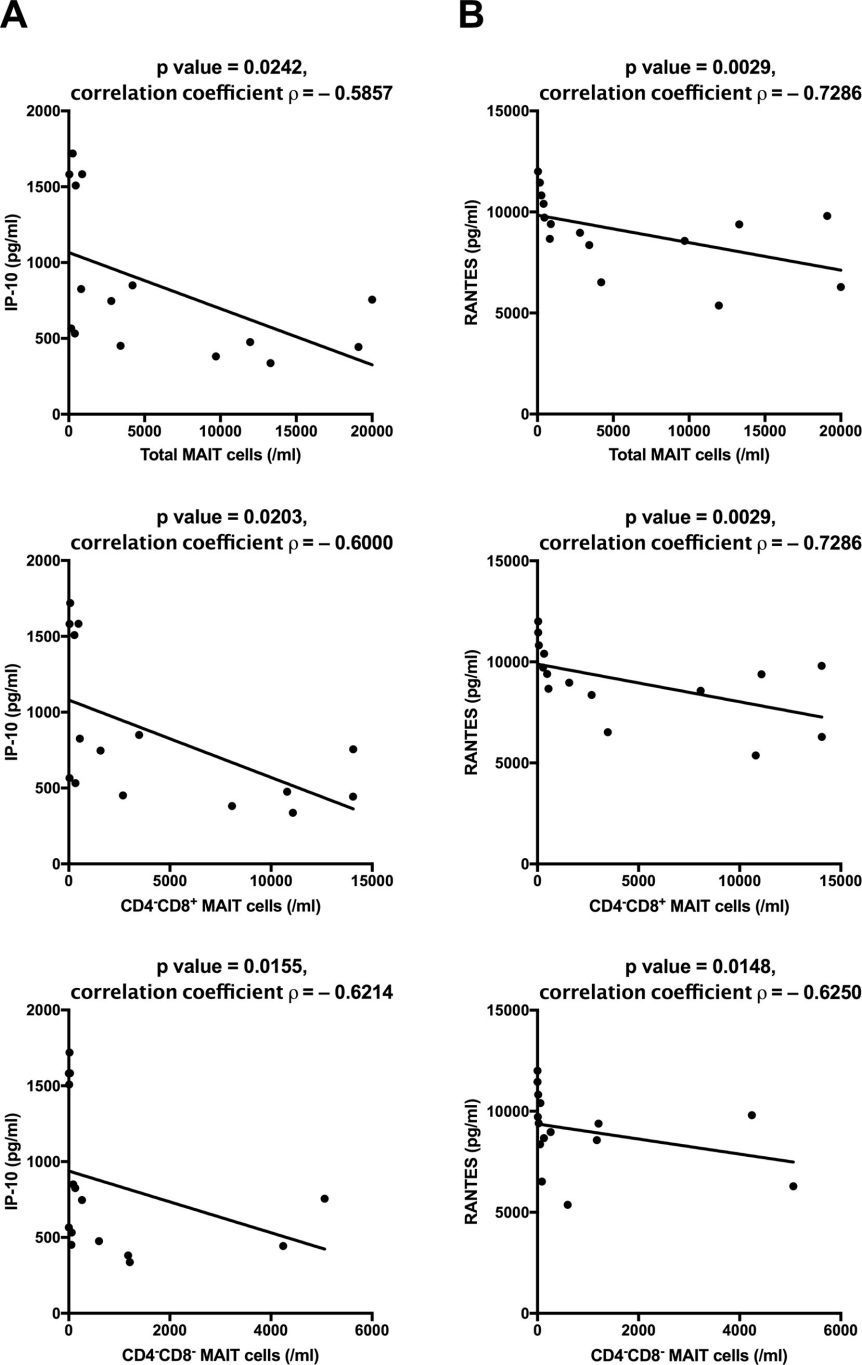
Correlations between the number of CD4^−^CD8^+^ MAIT cells in peripheral blood and the levels of cytokines in patients with ITP (*n* = 15). **(A)** Correlation between the number of total MAIT, CD4^−^CD8^+^ MAIT, and CD4^−^CD8^−^ MAIT cells and the concentration of IP-10 in the plasma of patients with ITP. IP-10 showed a negative correlation with the number of total MAIT and CD4^−^CD8^+^ MAIT cells. **(B)** Correlation between the number of total MAIT, CD4^−^CD8^+^ MAIT, CD4^−^CD8^−^ MAIT cells and the concentration of RANTES in the plasma of patients with ITP. RANTES also exhibited a negative correlation with the number of total MAIT and CD4^−^CD8^+^ MAIT cells. Spearman’s rank correlation coefficient was calculated, and hypothesis testing was performed to identify statistical significance.

We examined the concentration of 27 representative cytokines in the plasma of HCs and patients with ITP. As the result, IL-2, IL-5, IL-15, and GM-CSF were undetectable in all samples and we excluded them from the analysis. Then we analyzed the results according to the different groups and subgroups and tried to find correlations between cytokines and the number of MAIT cells. We found no significant differences in any cytokine level among HCs and ITP patients’ subgroups (no prednisolone treatment group, prednisolone responder group, and prednisolone non-responder group) (S5 Fig). Our analysis included only 3 HCs and 3 ITP patients with no prednisolone treatment, and the absence of significant results may be due to the lack of statistical power. In contrast, IP-10 and RANTES in patients with ITP showed a negative correlation with the number of total MAIT cells (*p* = 0.0242 and 0.0029, respectively), CD4^−^CD8^+^ MAIT cells (*p* = 0.0203 and 0.0029, respectively), and CD4^−^CD8^−^ MAIT cells (*p* = 0.0155 and 0.0148, respectively) (Fig 4A and 4B).

## Discussion

To the best of our knowledge, this study represents the first attempt at investigating the quantitative changes of MAIT cells in patients with ITP. We showed that the number of MAIT cells decreased in ITP patients and that these cells were especially reduced in non-responders to prednisolone treatment. Quantitative and functional deficiencies of MAIT cells in PB have been reported in infections and autoimmune diseases, including human HIV infection, tuberculosis, multiple sclerosis, systemic lupus erythematosus, rheumatoid arthritis, Crohn’s disease, ulcerative colitis, and autoimmune liver disease [4, 9, 10–15, 25, 26]. However, the role of MAIT cells in these diseases remains elusive. In the present study, we drew attention to the availability of MAIT cells as a surrogate marker for diagnosis and prognosis prediction in patients with ITP.

We examined the number of MAIT cells in PB of patients with ITP in a cross-sectional manner because the prevalence rate of ITP patients in the total population of Japan in 2013 was only 0.02%, and their incidence rate might be remarkably smaller than their prevalence rate. The blood samples were obtained from ITP patients with different history of the disease and treatment, and this was the limitation of our study. The effect of corticosteroids on MAIT cells is controversial. Most MAIT cells are noncycling. That is, <1% are Ki-67^+^, and they express the multidrug-resistance transporter (ABCB1), making them more resistant to various medications than other T-cell populations [1]. In fact, a correlation between the number of MAIT cells and use of corticosteroids, immunosuppressive agents, or disease-modifying antirheumatic drugs is not observed in patients with systemic lupus erythematosus [14]. In contrast, Hinks et al demonstrated that the frequency of MAIT cells was suppressed after 7 days of treatment with 20 mg oral prednisolone in patients with asthma [22–24]. Interestingly, the frequency of MAIT cells in patients with multiple sclerosis significantly increased 2–3 months after steroid therapy [10]. The reactivity of MAIT cells to corticosteroids may depend on comorbidities. In this study, we could not find any correlation between the number and frequency of MAIT cells and duration of prednisolone treatment in patients with ITP (S3 Fig). In addition, almost no change was noted in the number of total MAIT, CD4^−^CD8^+^ MAIT, and CD4^−^CD8^−^ MAIT cells in our two patients (S4 Fig). Based on this observation, at least in patients with ITP, the number of CD4^−^CD8^+^ MAIT cells may not be affected by prednisolone treatment, and it could be a stable marker of T-cell abnormalities.

Several studies have demonstrated that T-cell deregulation plays a role in ITP development, particularly CD4^+^ T cells that regulate antiplatelet antibody production. Patients with ITP have been shown to exhibit abnormalities of CD4^+^ cells, including Th1 cell polarization, Th2 cell overactivation, Th17 cell derangement, Treg cell down-regulation, and Th1/Th2 or Th17/Treg imbalances [27–32]. Moreover, the Th17 cell PB levels of newly diagnosed patients with ITP have been associated with responsiveness to corticosteroid treatment [20]. Corticosteroids are generally accepted as the first-line therapy for patients with ITP, with a response rate of approximately 60% [33]. Their key mechanism of action is considered to be their reduction in immunoglobulin production and antigen–antibody reactions, but the primary mechanism of corticosteroid resistance in patients with ITP is not clear.

Likewise, the mechanism of MAIT cell decrease is still unclear, but it is thought to be related, at least in part, to some T-cell abnormalities. Given the relationship between ITP and T-cell abnormalities, we are not surprised to see that ITP pathology correlates with MAIT cell profiles. In contrast, low number of MAIT cells does not correlate with the response to TPO-R agonist, the therapeutic agent against impaired platelet production, and this is also reasonable considering MAIT cells are not concerned with platelet generation. Booth et al. reported that the circulating MAIT cell frequency in *H*. *pylori*-infected volunteers (not ITP) was significantly lower than that in non-infected volunteers, and they hypothesized that MAIT in PB migrated into their infected gastric mucosa [34]. In our cases, there was no significant change in the circulating number of MAIT cells between *H*. *pylori*-positive ITP patients and *H*. *pylori*-negative ITP patients, and we considered that this was because low circulating number of MAIT cells in ITP patients resulted from T-cell abnormalities and not from reaction against foreign pathogens.

As described previously, we could not present the correlation between MAIT cells in the PB and prednisolone treatment in patients with ITP. Assuming that prednisolone treatment does not affect the number of MAIT cells in patients with ITP, clinicians can evaluate the number of MAIT cells irrespective of the treatment phase. However, this also indicates that the number of MAIT cells in patients with ITP may be consistently low after symptoms improved and treatment is completed. We believe that the number of MAIT cells in the PB of patients with ITP may reflect the severity of T-cell abnormalities and fragility of the immunoregulatory function, despite the fact that it is not correlated with the disease. If so, the number of CD4^−^CD8^+^ MAIT cells in the PB may predict the response to prednisolone in patients with ITP. Several effective second-line therapies are available for patients with ITP: rituximab, TPO-R agonist, and splenectomy. Thus, predicting prednisolone refractoriness in patients with ITP would allow clinicians to start second-line treatments at an early stage.

With regard to cytokines, researchers have shown that the plasma concentrations of Th-17-associated cytokines, IL-6, IL-17A, IL-17F, IL-22, and IL-23 are significantly higher in patients with ITP achieving CR by corticosteroid treatment than in HCs; however, these cytokines exhibited no significant difference between ITP patients with and without CR after corticosteroid treatment, and the authors concluded that the levels of these cytokines were not correlated with corticosteroid reactivity [20], a finding that agrees with ours. In addition, the level of cytokines generally changes due to effects of medication, infection, circadian variation, and others, so this is a fluctuating marker. To date, there is no evidence of a relationship between cytokines and the response to corticosteroids in patients with ITP. Meanwhile, we found that the plasma concentrations of IP-10 and RANTES in patients with ITP show a negative correlation with the number of CD4^−^CD8^+^ MAIT cells in our experiments. Mature MAIT cells derived from induced pluripotent stem cells (iPSCs) have been shown to produce a higher level of IP-10 (CXCL10) than immature cord blood-derived MAIT cells, and cord blood-derived MAIT cells exhibit enhanced production of RANTES (CCL5) compared with MAIT cells derived from iPSCs [35]. Considering MAIT cells’ protective effects against inflammation in a mouse intestinal inflammation model [11], quantitative and qualitative defects in MAIT cells and T-cell abnormalities may contribute to the enhanced production of inflammatory cytokines, but the relationship between MAIT cells and cytokines remains to be elucidated.

In conclusion, although further research, maybe multi-institutional joint research, is required to clarify the relation between ITP pathogenesis and MAIT cell alteration, the present study demonstrates that total MAIT and CD4^−^CD8^+^ MAIT cells in PB are significantly decreased in patients with ITP, correlating with their response to prednisolone treatment. The measurements of the number of total MAIT and CD4^−^CD8^+^ MAIT cells may lead to accurate diagnoses of ITP and may serve to predict the response to prednisolone treatment.

## Supporting information

S1 Fig Course of treatment and sample collection in individual patients with immune thrombocytopenia (ITP). Each symbol represents the event related to treatment, sample collection, and so on. TPO-R, thrombopoietin receptor.
(TIFF)

S2 Fig Representative plots of flow cytometry analysis showing the gating strategy of MAIT cells and their subsets. (A) To define MAIT cells, we started by gating lymphocyte subsets in FSC-A/SSC-A dot plot and by eliminating cell doublets in FSC-H/FSC-W dot plot. Subsequently, CD3^+^TCR-Vα7.2^+^ lymphocytes were gated, and then, the CD161^+^IL-18Rα^+^ subset was defined as MAIT cells. (B) To distinguish the CD4 CD8 subset of MAIT cells, we also started by gating lymphocyte subsets and then eliminating cell doublets in the same manner. Next, we gated CD161^+^TCR-Vα7.2^+^ lymphocytes and then divided them into CD4^+^CD8^+^, CD4^+^CD8^−^, CD4^−^CD8^−^, and CD4^−^CD8^+^ subsets.
(TIF)

S3 Fig Correlations between MAIT cells and duration of prednisolone treatment at the time of blood sample collection in patients with ITP. **(A)** Correlation between the number of total MAIT, CD4^−^CD8^+^ MAIT, and CD4^−^CD8^−^ MAIT cells as well as the duration of prednisolone treatment. No correlation was observed between the number of MAIT cell and duration of prednisolone treatment. **(B)** Correlation between the frequency of total MAIT, CD4^−^CD8^+^ MAIT, and CD4^−^CD8^−^ MAIT cells in the CD3^+^ T cells as well as the duration of prednisolone treatment. No correlation was observed between the frequency of MAIT cell and duration of prednisolone treatment. Spearman’s rank correlation coefficient was calculated, and hypothesis testing was conducted to identify statistical significance.
(TIFF)

S4 Fig Changes in the number of CD4^−^CD8^+^ MAIT cells in the peripheral blood of two patients with ITP after the initiation or discontinuation of corticosteroid treatment. (A) Changes in the number of total MAIT, CD4^−^CD8^+^ MAIT cells, and CD4^−^CD8^−^ MAIT cells in patients with ITP after the initiation of prednisolone treatment. Compared with the levels before the treatment, the number of total MAIT, CD4^−^CD8^+^ MAIT, and CD4^−^CD8^−^ MAIT cells did not vary significantly after the prednisolone induction. (B) Changes in the number of total MAIT, CD4^−^CD8^+^ MAIT, and CD4^−^CD8^−^ MAIT cells in patients with ITP after the termination of the prednisolone treatment. Twenty-four months after prednisolone discontinuation, the number of total MAIT, CD4^−^CD8^+^ MAIT, and CD4^−^CD8^−^ MAIT cells remained at extremely low levels.
(TIFF)

S5 Fig The concentration of cytokines in the peripheral blood of healthy controls (HCs) and patients with ITP. The concentration of IL-1ß, IL-1Ra, IL-4, IL-6, IL-7, IL-8, IL-9, IL-10, IL-12, IL-13, IL-17, Eotaxin, FGF, G-CSF, IFN-γ, IP-10, MCP-1, MIP-1α, PDGF-BB, MIP-1ß, RANTES, TNF-α, and VEGF in HCs (n = 3) and ITP patients (n = 15). ITP patients were divided into no prednisolone treatment group (n = 3), prednisolone responder group (n = 5) and prednisolone non-responder group (n = 7). There was no significant change in the concentration of all cytokines among the four groups. Statistical significance was calculated by the Steel–Dwass test.
(TIFF)

## References

[1] Dusseaux M, Martin E, Serriari N, Peguillet I, Premel V, Louis D, et al Human MAIT cells are xenobiotic-resistant, tissue-targeted, CD161hi IL-17-secreting T cells. Blood 2011; 117(4): 1250–1259. 10.1182/blood-2010-08-303339 21084709

[2] Le Bourhis L, Guerri L, Dusseaux M, Martin E, Soudais C, Lantz O. Mucosal-associated invariant T cells: unconventional development and function. Trends Immunol 2011; 32(5), 212–218. 10.1016/j.it.2011.02.005 21459674

[3] Kjer-Nielsen L, Patel O, Corbett AJ, Le Nours J, Meehan B, Liu L, et al MR1 presents microbial vitamin B metabolites to MAIT cells. Nature 2012; 491: 717–723. 10.1038/nature11605 23051753

[4] Le Bourhis L, Martin E, Peguillet I, Guihot A, Froux N, Core M, et al Antimicrobial activity of mucosal-associated invariant T cells. Nat Immunol 2010; 11(8): 701–708. 10.1038/ni.1890 20581831

[5] Ussher JE, Bilton M, Attwod E, Shadwell J, Richardson R, de Lara C, et al CD161++ CD8+ T cells, including the MAIT cell subset, are specifically activated by IL-12+IL-18 in a TCR-independent manner. Eur J Immunol 2014; 44(1), 195–203. 10.1002/eji.201343509 24019201 PMC3947164

[6] Le Bourhis L, Dusseaux M, Bohineust A, Bessoles S, Martin E, Premel V, et al MAIT cells detect and efficiently lyse bacterially-infected epithelial cells. PLoS Pathog 2013; 9(10): e1003681 10.1371/journal.ppat.1003681 24130485 PMC3795036

[7] Cosmi L, De Palma R, Santarlasci V, Maggi L, Capone M, Frosali F, et al Human interleukin 17-producing cells originate from a CD161+CD4+ T cell precursor. J Exp Med 2008; 205(8): 1903–1916. 10.1084/jem.20080397 18663128 PMC2525581

[8] Walker LJ, Kang YH, Smith MO, Tharmalingham H, Ramamurthy N, Fleming VM, et al Human MAIT and CD8alphaalpha cells develop from a pool of type-17 precommitted CD8+ T cells. Blood 2012; 119(2): 422–433. 10.1182/blood-2011-05-353789 22086415 PMC3257008

[9] Illes Z, Shimamura M, Newcombe J, Oka N, Yamamura T. Accumulation of Valpha7.2-Jalpha33 invariant T cells in human autoimmune inflammatory lesions in the nervous system. Int Immunol 2004; 16(2): 223–230. 14734607 10.1093/intimm/dxh018

[10] Miyazaki Y, Miyake S, Chiba A, Lantz O, Yamamura T. Mucosal-associated invariant T cells regulate Th1 response in multiple sclerosis. Int Immunol 2011; 23(9): 529–535. 10.1093/intimm/dxr047 21712423

[11] Ruijing X, Mengjun W, Xiaoling Z, Shu P, Mei W, Yingcheng Z, et al Jalpha33+ MAIT cells play a protective role in TNBS induced intestinal inflammation. Hepatogastroenterology 2012; 59(115): 762–767. 10.5754/hge11432 22115767

[12] Serriari NE, Eoche M, Lamotte L, Lion J, Fumery M, Marcelo P, et al Innate mucosal-associated invariant T (MAIT) cells are activated in inflammatory bowel diseases. Clin Exp Immunol 2014; 176(2), 266–274. 10.1111/cei.12277 24450998 PMC3992039

[13] Haga K, Chiba A, Shibuya T, Osada T, Ishikawa D, Kodani T, et al MAIT cells are activated and accumulated in the inflamed mucosa of ulcerative colitis. J Gastroenterol Hepatol 2016; 31(5): 965–972. 10.1111/jgh.13242 26590105

[14] Cho YN, Kee SJ, Kim TJ, Jin HM, Kim MJ, Jung HJ, et al Mucosal-associated invariant T cell deficiency in systemic lupus erythematosus. J Immunol 2014; 193(8): 3891–3901. 10.4049/jimmunol.1302701 25225673

[15] Bottcher K, Rombouts K, Saffioti F, Roccarina D, Rosselli M, Hall A, et al MAIT cells are chronically activated in patients with autoimmune liver disease and promote pro-fibrogenic hepatic stellate cell activation. Hepatology 2018 10.1002/hep.29782 29328499

[16] Bhattacharyya A, Hanafi LA, Sheih A, Golob JL, Srinivasan S, Boeckh MJ, et al Graft-derived reconstitution of mucosal-associated invariant T cells after allogeneic hematopoietic cell transplantation. Biol Blood Marrow Transplant 2018; 24(2): 242–251. 10.1016/j.bbmt.2017.10.003 29024803 PMC5806215

[17] Rodeghiero F, Stasi R, Gernsheimer T, Michel M, Provan D, Arnold DM, et al Standardization of terminology, definitions and outcome criteria in immune thrombocytopenic purpura of adults and children: report from an international working group. Blood 2009; 113(11): 2386–2393. 10.1182/blood-2008-07-162503 19005182

[18] Stasi R, Sarpatwari A, Segal JB, Osborn J, Evangelista ML, Cooper N, et al Effects of eradication of Helicobacter pylori infection in patients with immune thrombocytopenic purpura: a systematic review. Blood 2009; 113(6): 1231–1240. 10.1182/blood-2008-07-167155 18945961

[19] Johnsen J. Pathogenesis in immune thrombocytopenia: new insights. Hematology Am Soc Hematol Educ Program 2012; 2012: 306–312. 10.1182/asheducation-2012.1.306 23233597

[20] Zhang Y, Ma T, Zhou X, Chen J, Li J. Circulating level of Th17 cells is associated with sensitivity to glucocorticoids in patients with immune thrombocytopenia. Int J Hematol 2018 10.1007/s12185-017-2392-0 29327325

[21] Bajnok A, Berta L, Orban C, Veres G, Zadori D, Barta H, et al Distinct cytokine patterns may regulate the severity of neonatal asphyxia-an observational study. J Neuroinflammation 2017; 14(1), 244 10.1186/s12974-017-1023-2 29233180 PMC5727967

[22] Hinks TS, Zhou X, Staples K, Dimitrov BD, Manta A, Petrossian T et al Innate and adaptive T cells in ashtmatic patients: relationship to severity and disease mechanisms. J Allergy Clin Immunol 2015; 136: 323–333. 10.1016/j.jaci.2015.01.014 25746968 PMC4534770

[23] Hinks T, Zhou X, Staples K, Dimitrov BD, Manta A, Petrossian T et al Multidimensional endotypes of asthma: topological data analysis of cross-sectional clinical, pathological, and immunological data. Lancet 2015; 385(Suppl 1): S42. 10.1016/S0140-6736(15)60357-9 26312864

[24] Hinks TS. Mucosal-associated invariant T cells in autoimmunity, immune-mediated diseases and airways disease. Immunology 2016; 148(1): 1–12. 10.1111/imm.12582 26778581 PMC4819138

[25] Leeansyah E, Ganesh A, Quigley MF, Sonnerborg A, Andersson J, Hunt PW, et al Activation, exhaustion, and persistent decline of the antimicrobial MR1-restricted MAIT-cell population in chronic HIV-1 infection. Blood 2013; 121(7), 1124–1135. 10.1182/blood-2012-07-445429 23243281 PMC3575756

[26] Cosgrove C, Ussher JE, Rauch A, Gartner K, Kurioka A, Huhn MH, et al Early and nonreversible decrease of CD161++ /MAIT cells in HIV infection. Blood 2013; 121(6): 951–961. 10.1182/blood-2012-06-436436 23255555 PMC3567342

[27] Ji X, Zhang L, Peng J, Hou M. T cell immune abnormalities in immune thrombocytopenia. J Hematol Oncol 2014; 7: 72 10.1186/s13045-014-0072-6 25274611 PMC4189678

[28] Zhao Z, Yang L, Yang G, Zhuang Y, Qian X, Zhou X, et al Contributions of T lymphocyte abnormalities to therapeutic outcomes in newly diagnosed patients with immune thrombocytopenia. PLoS One 2015; 10(5), e0126601 10.1371/journal.pone.0126601 25978334 PMC4433177

[29] Hu Y, Wang X, Yu S, Hou Y, Ma D, Hou M. Neutralizations of IL-17A and IL-21 regulate regulatory T cell/T-helper 17 imbalance via T-helper 17-associated signaling pathway in immune thrombocytopenia. Expert Opin Ther Targets 2015; 19(6): 723–732. 10.1517/14728222.2015.1016499 25976230

[30] Ji L, Zhan Y, Hua F, Li F, Zou S, Wang W, et al The ratio of Treg/Th17 cells correlates with the disease activity of primary immune thrombocytopenia. PLoS One 2012; 7(12): e50909 10.1371/journal.pone.0050909 23226546 PMC3513316

[31] Liu H, Ouyang X, Li Y, Zeng H, Wang X, Xie S, et al Involvement of levels of Toll like receptor-4 in monocytes, CD4+ T-lymphocyte subsets, and cytokines in patients with immune thrombocytopenic purpura. Thromb Res 2013; 132(2): 196–201. 10.1016/j.thromres.2013.04.025 23830211

[32] Li J, Wang Z, Hu S, Zhao X, Cao L. Correction of abnormal T cell subsets by high-dose dexamethasone in patients with chronic idiopathic thrombocytopenic purpura. Immunol Lett 2013; 154(1–2): 42–48. 10.1016/j.imlet.2013.08.006 23994430

[33] Gudbrandsdottir S, Birgens HS, Frederiksen H, Jensen BA, Jensen MK, Kjeldsen L, et al Rituximab and dexamethasone vs dexamethasone monotherapy in newly diagnosed patients with primary immune thrombocytopenia. Blood 2013; 121(11). 1976–1981. 10.1182/blood-2012-09-455691 23293082

[34] Booth JS, Salerno-Goncalves R, Blanchard TG, Patil SA, Kader HA, Safta AM, et al Mucosal-Associated Invariant T Cells in the Human Gastric Mucosa and Blood: Role in Helicobacter pylori Infection. Front Immunol 2015; 6: 466 10.3389/fimmu.2015.00466 26441971 PMC4585133

[35] Wakao H, Yoshikiyo K, Koshimizu U, Furukawa T, Enomoto K, Matsunaga T, et al Expansion of functional human mucosal-associated invariant T cells via reprogramming to pluripotency and redifferentiation. Cell Stem Cell 2013; 12(5): 546–558. 10.1016/j.stem.2013.03.001 23523177

