# Supplementary figures and images for: Low mucosal-associated invariant T-cell number in peripheral blood of patients with immune thrombocytopenia and their response to prednisolone
Source: PLoS One. 2018 Nov 8;13(11):e0207149. doi: 10.1371/journal.pone.0207149 (PMC6224073; doi:10.1371/journal.pone.0207149)

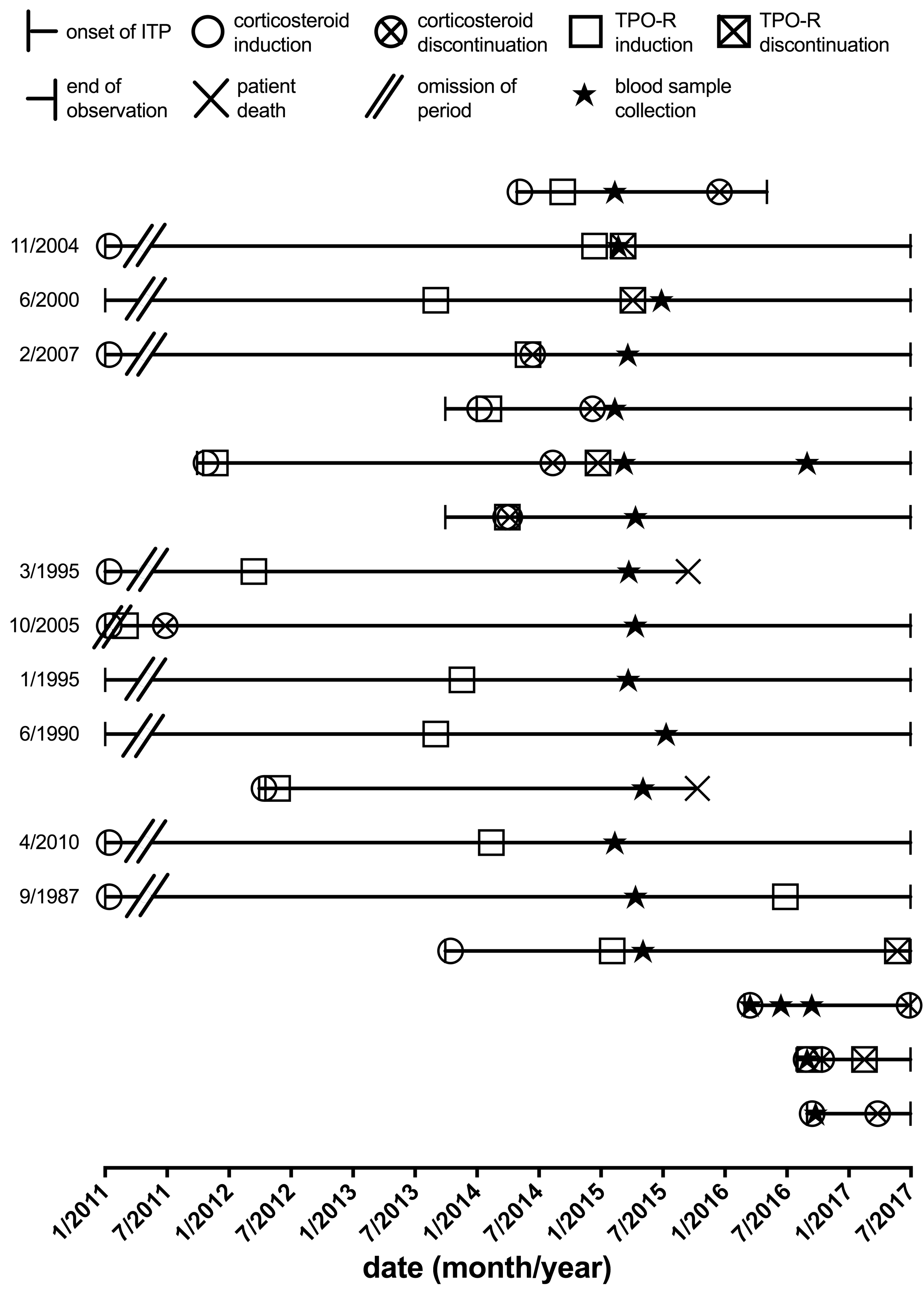

Supplement: S1 Fig — Each symbol represents the event related to treatment, sample collection, and so on. TPO-R, thrombopoietin receptor. (TIFF) [file pone.0207149.s001.tiff]

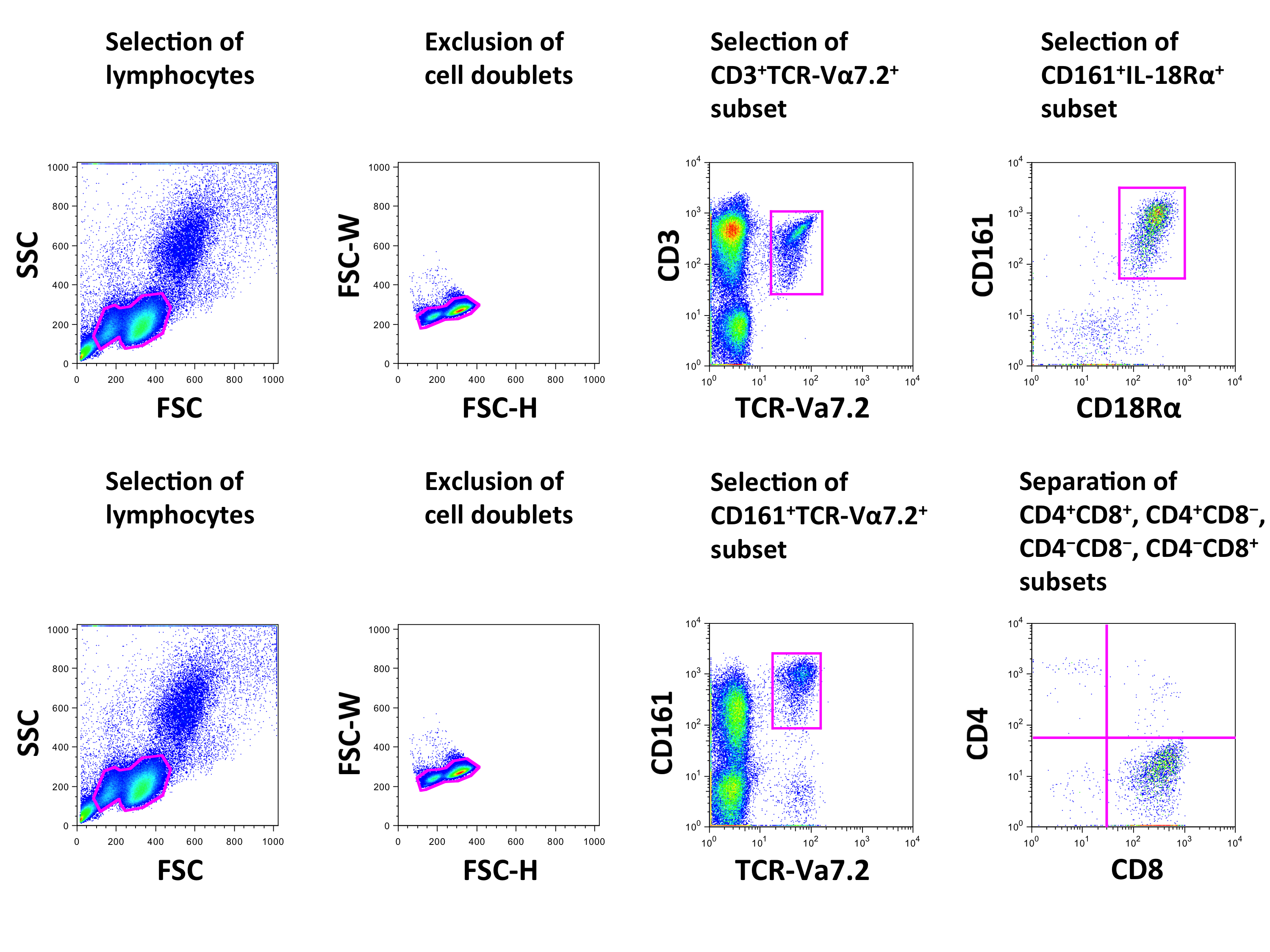

Supplement: S2 Fig — (A) To define MAIT cells, we started by gating lymphocyte subsets in FSC-A/SSC-A dot plot and by eliminating cell doublets in FSC-H/FSC-W dot plot. Subsequently, CD3+TCR-Vα7.2+ lymphocytes were gated, and then, the CD161+IL-18Rα+ subset was defined as MAIT cells. (B) To distinguish the CD4 CD8 subset of MAIT cells, we also started by gating lymphocyte subsets and then eliminating cell doublets in the same manner. Next, we gated CD161+TCR-Vα7.2+ lymphocytes and then divided them into CD4+CD8+, CD4+CD8−, CD4−CD8−, and CD4−CD8+ subsets. (TIF) [file pone.0207149.s002.tif]

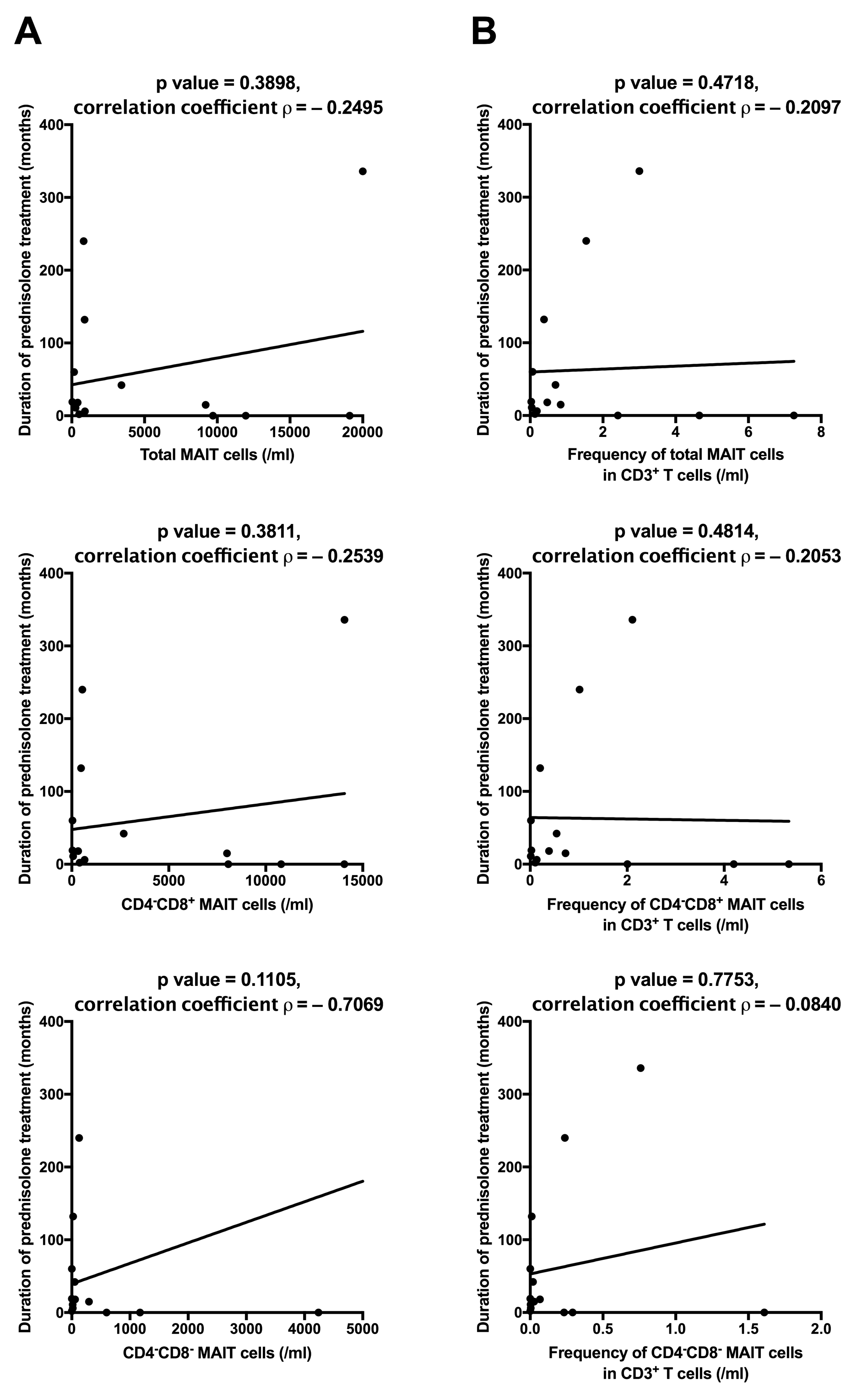

Supplement: S3 Fig — (A) Correlation between the number of total MAIT, CD4−CD8+ MAIT, and CD4−CD8− MAIT cells as well as the duration of prednisolone treatment. No correlation was observed between the number of MAIT cell and duration of prednisolone treatment. (B) Correlation between the frequency of total MAIT, CD4−CD8+ MAIT, and CD4−CD8− MAIT cells in the CD3+ T cells as well as the duration of prednisolone treatment. No correlation was observed between the frequency of MAIT cell and duration of prednisolone treatment. Spearman’s rank correlation coefficient was calculated, and hypothesis testing was conducted to identify statistical significance. (TIFF) [file pone.0207149.s003.tiff]

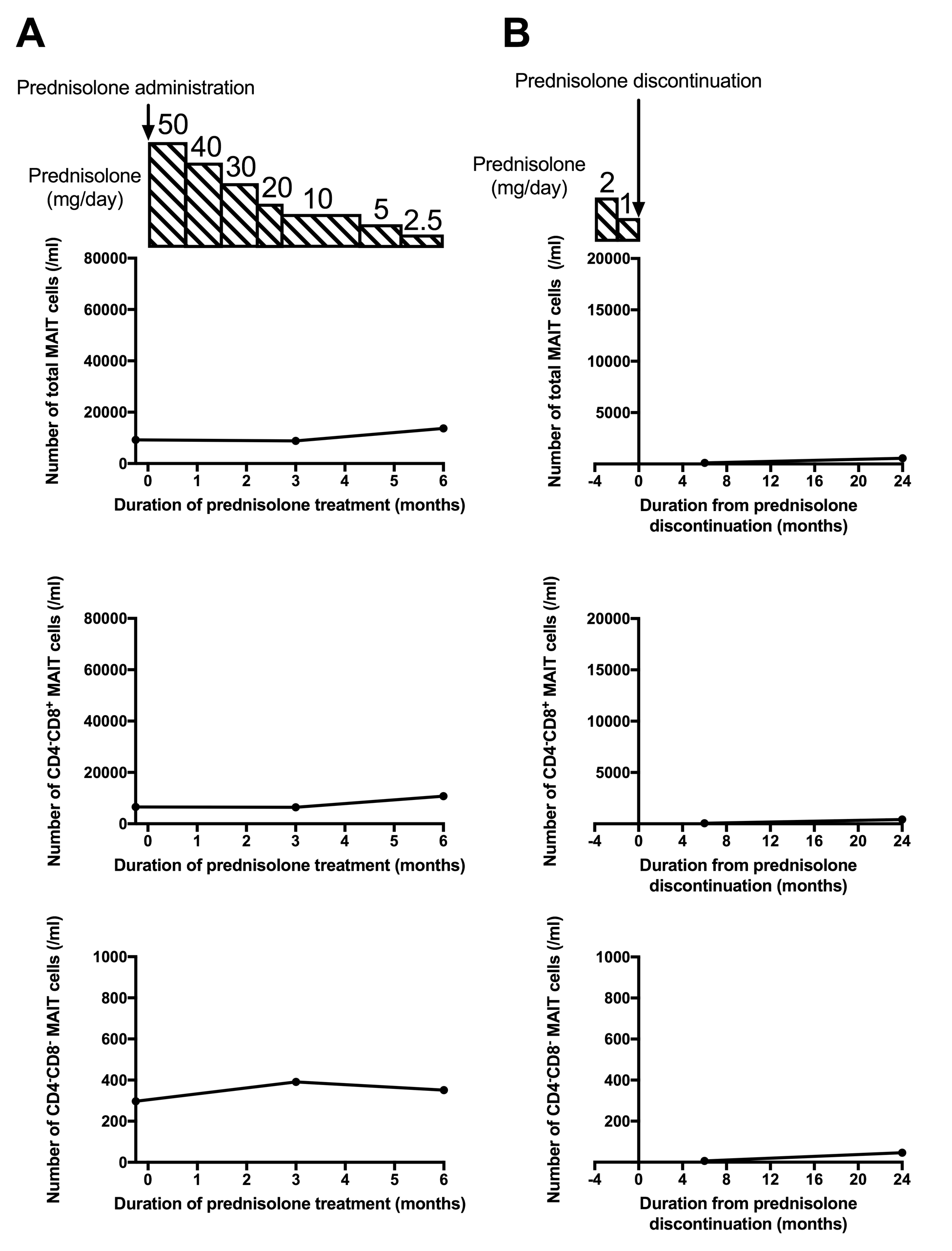

Supplement: S4 Fig — (A) Changes in the number of total MAIT, CD4−CD8+ MAIT cells, and CD4−CD8− MAIT cells in patients with ITP after the initiation of prednisolone treatment. Compared with the levels before the treatment, the number of total MAIT, CD4−CD8+ MAIT, and CD4−CD8− MAIT cells did not vary significantly after the prednisolone induction. (B) Changes in the number of total MAIT, CD4−CD8+ MAIT, and CD4−CD8− MAIT cells in patients with ITP after the termination of the prednisolone treatment. Twenty-four months after prednisolone discontinuation, the number of total MAIT, CD4−CD8+ MAIT, and CD4−CD8− MAIT cells remained at extremely low levels. (TIFF) [file pone.0207149.s004.tiff]

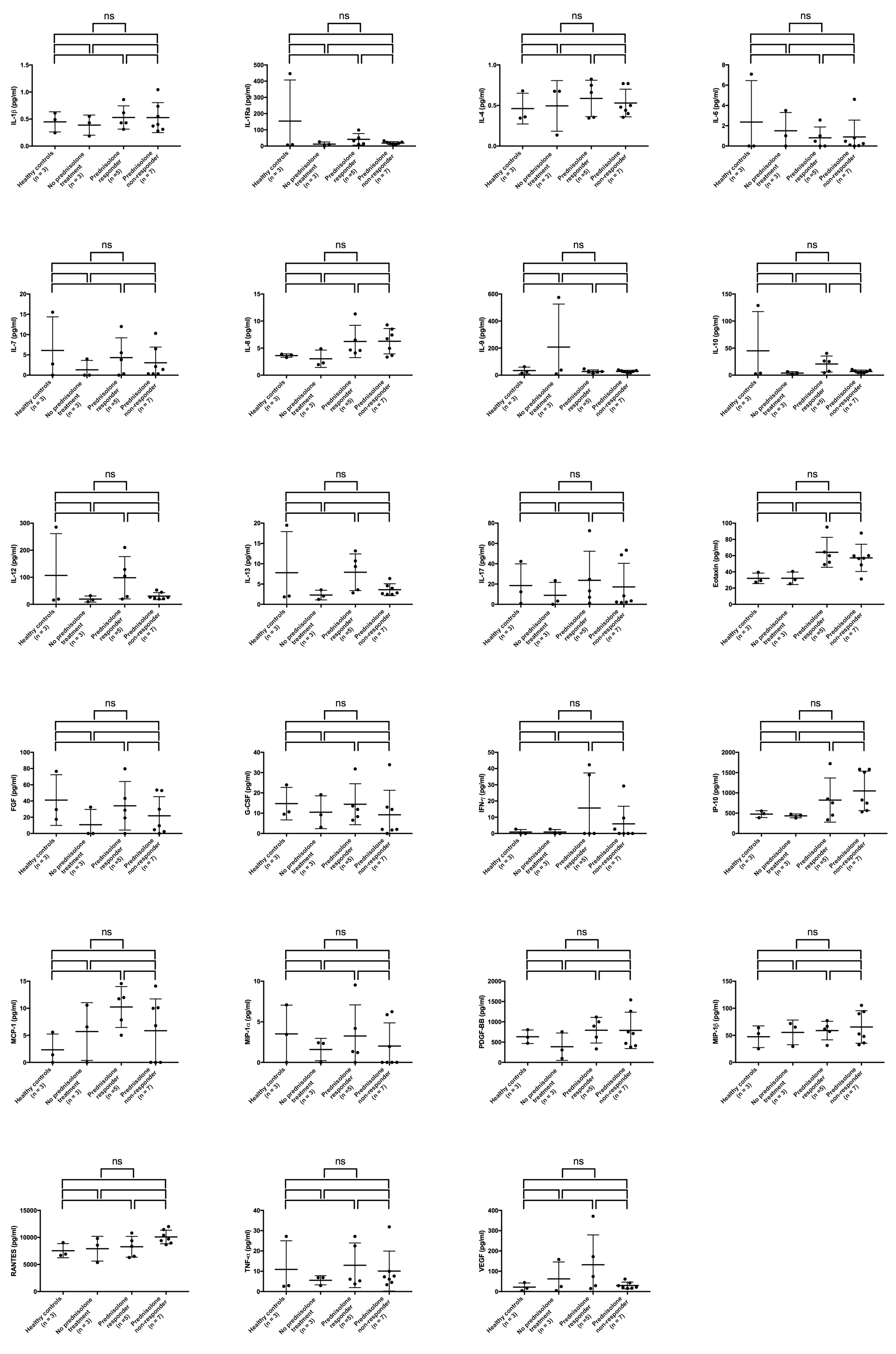

Supplement: S5 Fig — The concentration of IL-1ß, IL-1Ra, IL-4, IL-6, IL-7, IL-8, IL-9, IL-10, IL-12, IL-13, IL-17, Eotaxin, FGF, G-CSF, IFN-γ, IP-10, MCP-1, MIP-1α, PDGF-BB, MIP-1ß, RANTES, TNF-α, and VEGF in HCs (n = 3) and ITP patients (n = 15). ITP patients were divided into no prednisolone treatment group (n = 3), prednisolone responder group (n = 5) and prednisolone non-responder group (n = 7). There was no significant change in the concentration of all cytokines among the four groups. Statistical significance was calculated by the Steel–Dwass test. (TIFF) [file pone.0207149.s005.tiff]
